# Colon Cancer Tumorigenesis Initiated by the H1047R Mutant PI3K

**DOI:** 10.1371/journal.pone.0148730

**Published:** 2016-02-10

**Authors:** Alexander E. Yueh, Susan N. Payne, Alyssa A. Leystra, Dana R. Van De Hey, Tyler M. Foley, Cheri A. Pasch, Linda Clipson, Kristina A. Matkowskyj, Dustin A. Deming

**Affiliations:** 1 Division of Hematology and Oncology, Department of Medicine, University of Wisconsin School of Medicine and Public Health, Madison, WI, United States of America; 2 University of Wisconsin Carbone Cancer Center, Madison, WI, United States of America; 3 Department of Oncology, University of Wisconsin Madison, Madison, WI, United States of America; 4 Department of Pathology and Laboratory Medicine, University of Wisconsin School of Medicine and Public Health, Madison, WI, United States of America; 5 William S Middleton Memorial Veterans Hospital, Madison, WI, United States of America; Cedars-Sinai Medical Center, UNITED STATES

## Abstract

The phosphoinositide 3-kinase (PI3K) signaling pathway is critical for multiple important cellular functions, and is one of the most commonly altered pathways in human cancers. We previously developed a mouse model in which colon cancers were initiated by a dominant active PI3K p110-p85 fusion protein. In that model, well-differentiated mucinous adenocarcinomas developed within the colon and initiated through a non-canonical mechanism that is not dependent on WNT signaling. To assess the potential relevance of PI3K mutations in human cancers, we sought to determine if one of the common mutations in the human disease could also initiate similar colon cancers. Mice were generated expressing the *Pik3ca*^*H1047R*^ mutation, the analog of one of three human hotspot mutations in this gene. Mice expressing a constitutively active PI3K, as a result of this mutation, develop invasive adenocarcinomas strikingly similar to invasive adenocarcinomas found in human colon cancers. These tumors form without a polypoid intermediary and also lack nuclear CTNNB1 (β-catenin), indicating a non-canonical mechanism of tumor initiation mediated by the PI3K pathway. These cancers are sensitive to dual PI3K/mTOR inhibition indicating dependence on the PI3K pathway. The tumor tissue remaining after treatment demonstrated reduction in cellular proliferation and inhibition of PI3K signaling.

## Introduction

Colon cancer is a heterogeneous disease with multiple subtypes distinguished by their mutation profiles. Tumorigenesis is commonly thought to arise in the colon secondary to the sequential acquisition of mutations within the epithelial cells lining the base of the crypt. Alterations in the *Adenomatous Polyposis Coli* (*APC*) tumor suppressor gene are thought to be the initiating event in the majority of sporadic human colorectal cancers with approximately 80–90% of human colon cancers harboring somatic mutations in *APC* [1–3]. Following loss of APC, tumors develop within the colon largely through the canonical adenoma-to-carcinoma sequence [4]. During this process the premalignant adenoma acquires mutations in other genes including *KRAS*, *TP53*, *PIK3CA*, and *BRAF* resulting in the eventual formation of an invasive adenocarcinoma and diversity in the molecular profile [5]. Non-canonical mechanisms of colon cancer initiation have also been described including the serrated polyp-carcinoma pathway [6].

The *PIK3CA* gene encodes for the p110 alpha catalytic subunit of PI3K and mutations occur in 20–30% of human colorectal cancers [7, 8]. There are three hotspot mutations that occur in *PIK3CA*: E542K, E545K, and H1047R, all resulting in a constitutively active PI3K [9]. We previously developed a transgenic mouse model expressing a constitutively active PI3K throughout the distal small intestine and colon (*Fc*^*+*^ *Pik3ca*^p110*^) [10]. These mice developed invasive mucinous adenocarcinomas in the proximal colon. These cancers frequently invaded through the muscularis propria to the serosal surface. Cancer was also detected in regional lymph nodes and as satellite tumor deposits in the adjacent adipose tissue. Nuclear localization of CTNNBI (β-catenin) was not identified within these tumors, indicating that the WNT signaling cascade is not aberrant as would be expected if these cancers developed through the canonical adenoma-to-carcinoma pathway. These tumors also developed without an identifiable polypoid precursor lesion. While the *Fc*^*+*^ *Pik3ca*^p110*^ model results in a constitutively active PI3K, it uses a p85-p110 fusion protein not found in human cancers. Here we investigate whether tumorigenesis in the colon can be initiated secondary to the H1047R hotspot mutation in *Pik3ca* (*Pik3ca*^*H1047R*^).

## Materials and Methods

### Mouse Husbandry

All animal studies were conducted under protocols approved by the Institutional Animal Care and Use Committee at the University of Wisconsin–Madison, following the guidelines of the American Association for the Assessment and Accreditation of Laboratory Animal Care. Homozygous FVB.*Fc*^*+*^ female mice (NCI Mouse Repository; Strain Number 01XD8) were crossed to homozygous *Pik3ca* ^*H1047R*^ male mice (The Jackson Laboratory; Stock Number 016977) to generate *Fc*^*+*^ *Pik3ca*^*H1047R*^ mice used in this study. Mice were genotyped for *Fc* and *Pik3ca*^*H1047R*^ as described previously [11, 12]. *Apc*^*Min/+*^ mice (C57BL/6J *Apc*^*Min*^/J; The Jackson Laboratory; Stock Number 002020) were maintained as previously described [13].

### Histology and Immunohistochemistry

At necropsy, the colon was removed, flushed with PBS, opened lengthwise and fixed in 10% buffered formalin for 24 hours. Tissues were then stored in 70% ethanol, processed, embedded in paraffin, and cut into 5 μm sections. Every tenth section was stained with hematoxylin and eosin (H&E) for histological review. Immunohistochemistry was performed by dewaxing and rehydrating paraffin-embedded tissues before antigen unmasking was carried out by boiling the samples for 35 minutes in citrate buffer (pH 6.0). Next a peroxidase quench (Peroxidazed 1, PX9684, BioCare Medical, Concord, CA) and background block (Background Sniper, BS966H, BioCare Medical) were applied before the primary antibody was left on overnight. The secondary antibody (Mach 2 Rabbit HRP-Polymer, RHRP520H, BioCare Medical) was applied for 30 minutes followed by chromogen staining (Betazoid DAB Chromogen Kit, BDB2004H, BioCare Medical). Slides were counterstained with hematoxylin (CAT Hematoxylin, CATHE-H, BioCare Medical) and dehydrated for mounting. The primary antibodies included: anti-pAKT (Ser473) (D9E) (#4060, 1:100, Cell Signaling Technology, Beverly, MA), anti-pRPS6 (Ser 235/236) (#4858, 1:50, Cell Signaling Technology), anti-Ki67 (D3B5) (#12202, 1:400, Cell Signaling Technology) and mouse anti-β-catenin (D10A8) (#8480, 1:200, Cell Signaling Technology). The Ki67 proliferation index was measured as the percent of nuclei staining positive for Ki67 per tumor using ImmunoRatio, an ImageJ plugin (http://jvsmicroscope.uta.fi/sites/default/files/software/immunoratio-plugin/index.html).

### Immunoblotting

Tissue samples were excised and flash frozen. After 24 hours, the samples were sonicated in T-PER tissue protein extraction reagent (Thermo Scientific, Pittsburg, PA), protease inhibitor cocktail (Sigma-Aldrich, St. Louis, MO), and phenylmethylsulfonyl fluoride (PMSF, Sigma-Aldrich). Extracted protein was then run as previously described [14]. Primary antibodies against pAKT (Ser473) (D9E) (#4060, Cell Signaling Technology), AKT (#4691, Cell Signaling Technology), pRPS6 (Ser235/236) (#4858, Cell Signaling Technology), total RPS6 (5G10) (#2217, Cell Signaling Technology), p4EBP1 (Thr37/46) (236B4) (#2855, Cell Signaling Technology), 4EBP1 (53H11) (#9644 Cell Signaling Technology) were incubated in 5% bovine serum albumin (Sigma-Aldrich) at a 1:1000 ratio for 16 hours. Anti-β-actin (#5125, Cell Signaling Technology) was utilized as a loading control at a ratio of 1:1000.

### Animal Treatment

*Fc*^*+*^ *Pik3ca*^*H1047R*^ mice over 150 days of age were selected and randomly assigned to the treatment and control groups. Baseline dual hybrid ^18^F-FDG PET/CT scans were performed prior to and 15 days following treatment initiation. Animals in the control arm received hydroxyethyl cellulose dissolved in water to a 1% final concentration by oral gavage daily for 14 days. Animals randomized to the NVP-BEZ235 arm received 35 mg/kg of NVP-BEZ235 dissolved in 1% hydroxyethyl cellulose [15]. For therapeutic investigations, necropsy was performed following 14 days of treatment.

### Dual hybrid ^18^F-Fluorodeoxyglucose (FDG) Positron Emission Tomography (PET)/Computed Tomography (CT) Imaging

Animals were fasted for at least 6 hours prior to injection of ^18^F-FDG (100 μCi; IBA Molecular, Romeoville, IL). After injection, the animals were kept under anesthesia for 60 minutes and then prepared for virtual colonography as described previously [16]. A PET acquisition was performed, followed immediately by CT scanning. Maximum intensity projections were created in Siemens Inveon Research Workplace (Knoxville, TN). The PET images were reconstructed using OSEM3D/MAP (OSEM3D, 2 iterations; MAP 18, iterations 16 subsets). Attenuation correction was performed using the CT data. The CT images were reconstructed using standard conebeam reconstruction. Baseline and post-treatment PET scans were normalized to injected dose, dose decay, activity, and weight. Tumor volumes were estimated from measurements on the PET/CT scans. PET imaging was utilized to locate tumors prior to volume estimation. Tumor volumes can only be estimated, as delineating the exact tumor boundaries is difficult. This is because many of these cancers are not luminal and subtle FDG signal changes related to the hyperplastic normal epithelium surrounding the tumors exist. Tumor volumes in each cohort were compared using a two-sided Wilcoxon rank sum test. A p-value of less than 0.05 was considered statistically significant.

## Results

**Fig 1:**
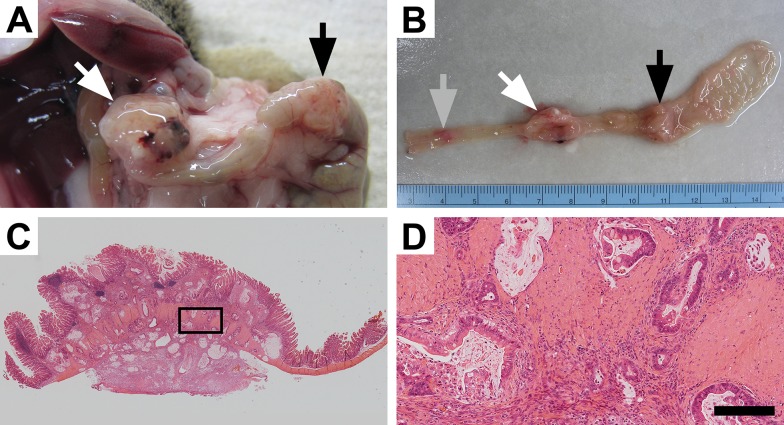
Mutant PI3K can lead to colon cancer development. Approximately 60% of *Fc*^*+*^ *Pik3ca*^*H1047R*^ mice develop tumors within the colon which can result in these mice becoming moribund. At necropsy large colon tumors are found extending through the colonic wall (A). Upon resection, multiple lesions can be identified within the colon and can be over 1 cm in size (A and B). Following histological sectioning, H&E staining demonstrates that these lesions are invasive mucinous adenocarcinomas of the colon without a predominant intra-luminal component (C). Higher magnification demonstrates an abundant desmoplastic reaction with surrounding mucin lakes lined with epithelial cancer cells (D). D size bar = 200 μm.

***Fc***^***+***^ ***Pik3ca***^***H1047R***^ **mice develop invasive cancers in the colon**: To determine if the *Pik3ca* H1047R hotspot mutation could initiate colon tumorigenesis within the colon, a set of 33 (16 male and 17 female) *Fc*^*+*^ *Pik3ca*^*H1047R*^ mice was generated. These mice express the mutant PI3K in the distal small intestine and colon secondary to the FABP1-Cre. At necropsy, the mice had a median age of 165 days, ranging from 97 to 310 days. Six mice were moribund at a median age of 193 days (range 97–310). All *Fc*^*+*^ *Pik3ca*^*H1047R*^ mice demonstrated hyperplasia of the colon and small intestine. Tumors within the colon were identified in 20 of the 33 mice (61%). Of the mice with tumors, the median number of tumors was 2 (range 1–6). Multiple mice developed tumors over 1 cm in size (Fig 1A and 1B). No metastatic disease or small intestinal cancers were identified in these mice. S1 Table compares the characteristics of the *Fc*^*+*^ *Pik3ca*^*H1047R*^ mice with those of the *Fc*^+^ Pik3c*a*^p110*^ and the *Apc*^*Min/+*^ mice. Following histological sectioning, these tumors were found to be invasive mucinous adenocarcinomas (Fig 1C and 1D). They often penetrated through the muscularis propria and extended to the serosa. On histological examination the tumors were flat rather than polypoid in morphology.

**Fig 2:**
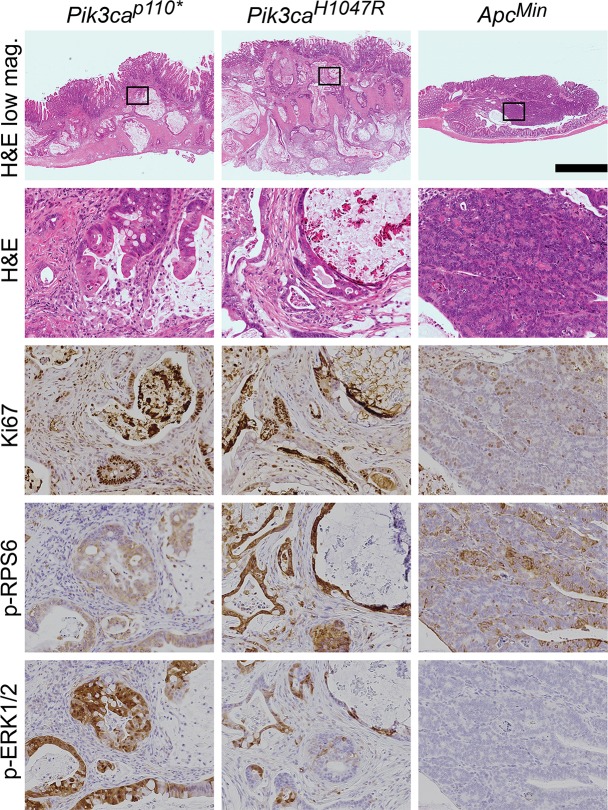
*Fc*^*+*^ *Pik3ca*^*H1047R*^ colon cancers are similar to those in *Fc*^*+*^ *Pik3c*a^p110*^ mice. In both of these *Pik3ca* mutant models, deeply invasive cancers are seen with the vast majority of the tumors having penetrated below the muscularis mucosa. This is in contrast to *Apc*^*Min*^ colon tumors, which typically are adenomatous tumors with no or just superficial invasion. Abundant mucin is present within both the *Fc*^*+*^ *Pik3ca*^*H1047R*^ and *Fc*^*+*^ *Pik3c*a^p110*^ colon cancers. These *Pik3ca* mutant cancers also demonstrate increased proliferation, as measured by nuclear Ki67, in comparison to *Apc*^*Min*^ tumors. In addition, phosphorylated RPS6 and ERK1/2 are increased above that seen in the *Apc*^*Min*^ colon lesions. Scale bar for low magnification images = 1mm. Enlargements are 10x magnifications of the areas outlined in the low magnification images. Min, *Apc*^*Min*^.

The *Fc*^*+*^ *Pik3ca*^*H1047R*^ cancers are histologically similar to those in *Fc*^*+*^ Pik3c*a*^p110*^ mice (Fig 2). The tumors from the *Fc*^+^ *Pik3ca*^*H1047R*^ and *Fc*^+^ Pik3c*a*^p110*^ animals exhibited deeply invasive mucinous adenocarcinomas without evidence of a precursor lesion. The tumors were not associated with epithelial surface dysplasia. In contrast, *Apc*^*Min*^ tumors developed from a dysplastic, polypoid precursor (tubular adenoma) and progressed to advanced adenomas (tubular adenoma with high-grade dysplasia) with rare progression to superficially invasive, well-differentiated adenocarcinomas.

*Fc*^*+*^ *Pik3ca*^*H1047R*^ cancers have increased cellular proliferation (Fig 2). Immunohistochemistry showed an increased Ki67 proliferative index in *Fc*^*+*^ *Pik3ca*^*H1047R*^ compared to *Apc*^*Min*^ tumors. *Fc*^*+*^ *Pik3ca*^*H1047R*^ tumors exhibited a proliferative index of >70% with most neoplastic nuclei exhibiting Ki67 nuclear expression. In contrast, *Apc*^*Min*^ tumors had a low proliferative index as demonstrated by less than 25% nuclear expression of nuclear Ki67.

***Pik3ca***^***H1047R***^ **results in activation of the PI3K/AKT/mTOR pathway and ERK 1/2 signaling:** The epithelial cells in *Fc*^*+*^ *Pik3ca*^*H1047R*^ and *Fc*^*+*^ Pik3c*a*^p110*^ colon cancers demonstrate increased staining for phosphorylation of RPS6 compared to those in *Apc*^*Min*^ colon tumors (Fig 2). In addition, an increase in phosphorylated ERK1/2 was observed in the *Pik3ca* mutant cancers (Fig 2).

**Fig 3:**
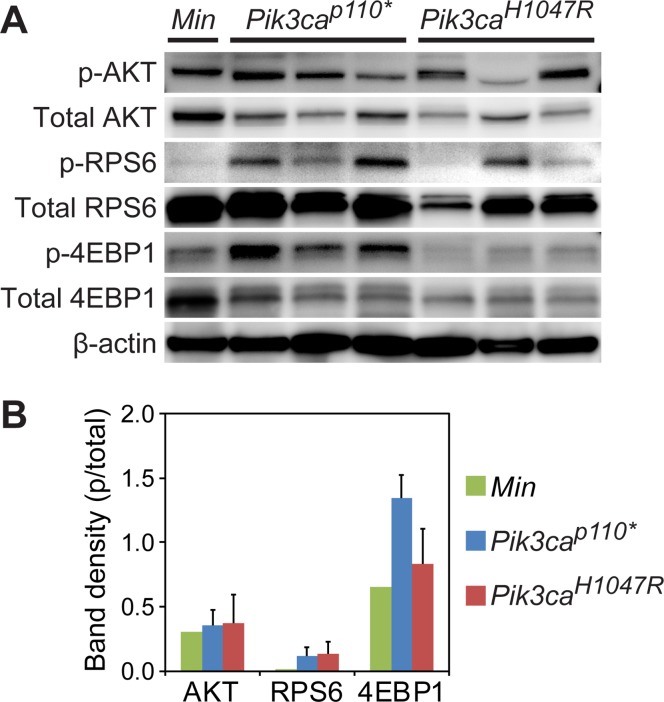
The PI3K pathway is activated in *Apc*^*Min/+*^, *Fc*^*+*^ *Pik3c*a^p110*^, and *Fc*^*+*^ *Pik3ca*^*H1047R*^ mice. Immunoblotting demonstrates robust phosphorylation of AKT in *Apc*^*Min*^, *Fc*^*+*^ *Pik3c*a^p110*^ and *Fc*^*+*^ *Pik3ca*^*H1047R*^ colon tumors (A). Increased phosphorylation of RPS6 and 4EBP1 beyond that seen in the *Apc*^*Min*^ lesions is observed in the *Fc*^*+*^ *Pik3ca*^*H1047R*^ and *Fc*^*+*^ *Pik3c*a^p110*^ colon tumors. The more aggressive phenotype seen in the *Fc*^*+*^ *Pik3c*a^p110*^ mice, with a greater number and decreased latency, is associated with an increased phosphorylation of 4EBP1 compared to *Fc*^*+*^ *Pik3ca*^*H1047R*^ tumors (B).

Immunoblotting demonstrated activation of the PI3K signaling cascade across tumor types (Fig 3A). A trend towards an increase in phosphorylation of AKT, RPS6, and 4EBP1 was seen in the *Fc*^*+*^ *Pik3ca*^*H1047R*^ and *Fc*^*+*^ *Pik3ca*^p110*^ cancers (Fig 3B). In comparison to *Fc*^*+*^ Pik3c*a*^p110*^ colon tumors, Fc^*+*^ *Pik3ca*^*H1047R*^ demonstrated a trend towards less phosphorylation of 4EBP1 (Fig 3B).

**Fig 4:**
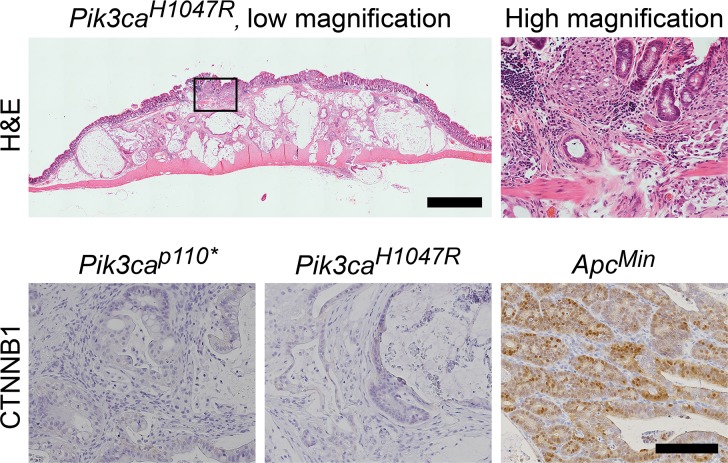
*Fc*^*+*^ *Pik3ca*^*H1047R*^ colon cancers develop through a non-canonical pathway. Histological examination demonstrates that these cancers are flat without a polypoid component. Low grade dysplasia (tubular adenoma) has not been identified in these or the *Fc*^*+*^ *Pik3c*a^p110*^ mice. At higher magnification, malignant glands above the muscularis mucosa can be identified which appear to be originating from the crypt bases without identification of surface low grade dysplasia. CTNNB1 staining demonstrates that nuclear CTNNB1 is absent in both of the *Pik3ca* mutant models, but is present in the *Apc*^*Min*^ controls. Scale bar for low magnification image = 1mm. High magnification H&E image is a 6x magnification of the area indicated in the image to the left. Scale bar for CTNNB1 images = 100μm.

***Pik3ca***^***H1047R***^ **mutations initiate cancers through a non-canonical mechanism**: The most common mutation in human colorectal adenocarcinomas results in a truncated APC protein. Truncation of the APC protein results in the loss of tumor suppression. A commonly used marker for APC function is ß-catenin translocation to the nucleus of the colonic epithelial cells. Immunohistochemistry of the tumor cells showed that nuclear localization of ß-catenin was absent in both the *Pik3ca*^*H1047R*^ and *Pik3ca*^*p110*^ cancers, but was abundantly present in the *Apc*^*Min*^ colon tumors (Fig 4). The lack of nuclear ß-catenin indicates that APC loss has not occurred. If these cancers were arising though the canonical mechanism of tumorigenesis, loss of APC would be expected, thus these tumors are developing through a non-canonical mechanism.

**Fig 5:**
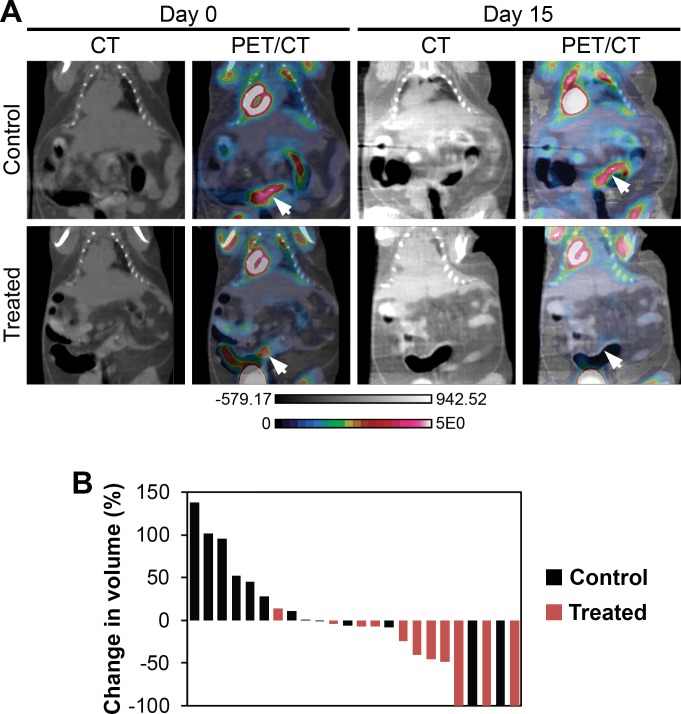
Dual PI3K/mTOR inhibition induces treatment responses in *Fc*^*+*^ *Pik3ca*^*H1047R*^ colon cancers. *Fc*^*+*^ *Pik3ca*^*H1047R*^ mice were treated with NVP-BEZ235 (35mg/kg/day) or control once daily by oral gavage for 14 days. These mice underwent dual hybrid 18F-FDG PET/CT imaging at baseline and then 24 hours following the last dose of study drug (A, arrows denote tumors pre- and post-treatment). A significant reduction in tumor volume, as measured on the CT images, was detected in the NVP-BEZ235 group (p = 0.008; B). In addition, there was a trend for a slight decrease in the median SUV for those cancers treated with the PI3K/mTOR inhibitor. In one control mouse, two tumors were observed on baseline imaging, but not detected on follow-up PET/CT imaging.

***Fc***^***+***^ ***Pik3ca***^***H1047R***^ **cancers are sensitive to dual PI3K/mTOR inhibition**: A second set of *Fc*^*+*^ *Pik3ca*^*H1047R*^ mice at 150 days of age was imaged with dual hybrid ^18^F-FDG microPET/CT. After 24 hours, 11 mice (4 male and 7 female) were initiated on NVP-BEZ235 at 35mg/kg/day in 1% hydroxycellulose by oral gavage daily and 8 mice (4 male and 4 female) were administered vehicle-only control. After 14 days of treatment, the mice were imaged again with PET/CT 24 hours after the last dose of NVP-BEZ235 or control. Images were normalized to injected dose of ^18^F-FDG, dose decay, activity, and mouse weight. Tumor volume was measured using the CT images acquired. The initial tumor volumes of treated and control mice were comparable (S2 Table). The percent change in tumor volume was calculated for each tumor identified on baseline imaging (Fig 5A and S1 Fig). A median increase in tumor volume of 11% was seen in the controls and a median reduction in tumor volume of 41% was observed in the cancers treated with NVP-BEZ235 (p = 0.01, two-sided Wilcoxon rank sum; Fig 5B). In one control mouse two tumors were able to be identified on baseline imaging, but not detected on the second scan (Fig 5B). The change in metabolic activity of each tumor was also assessed with ^18^F-FDG PET imaging. The median relative tumor avidity (avidity at day 15 divided by avidity at day 0) was 77% for cancers treated with NVP-BEZ235 compared to controls (p = 0.13, two-sided Wilcoxon rank sum test). The delay of 24 hours between NVP-BEZ235 dosing and PET scanning allows for the direct metabolic inhibition secondary to this agent to resolve. The changes in avidity are thus related to changes in tumor size and activity. These changes in avidity are also likely an underestimate of the change secondary to this treatment, as a tumor flare has been described following the withdrawal of mTOR inhibitors [17].

**Fig 6:**
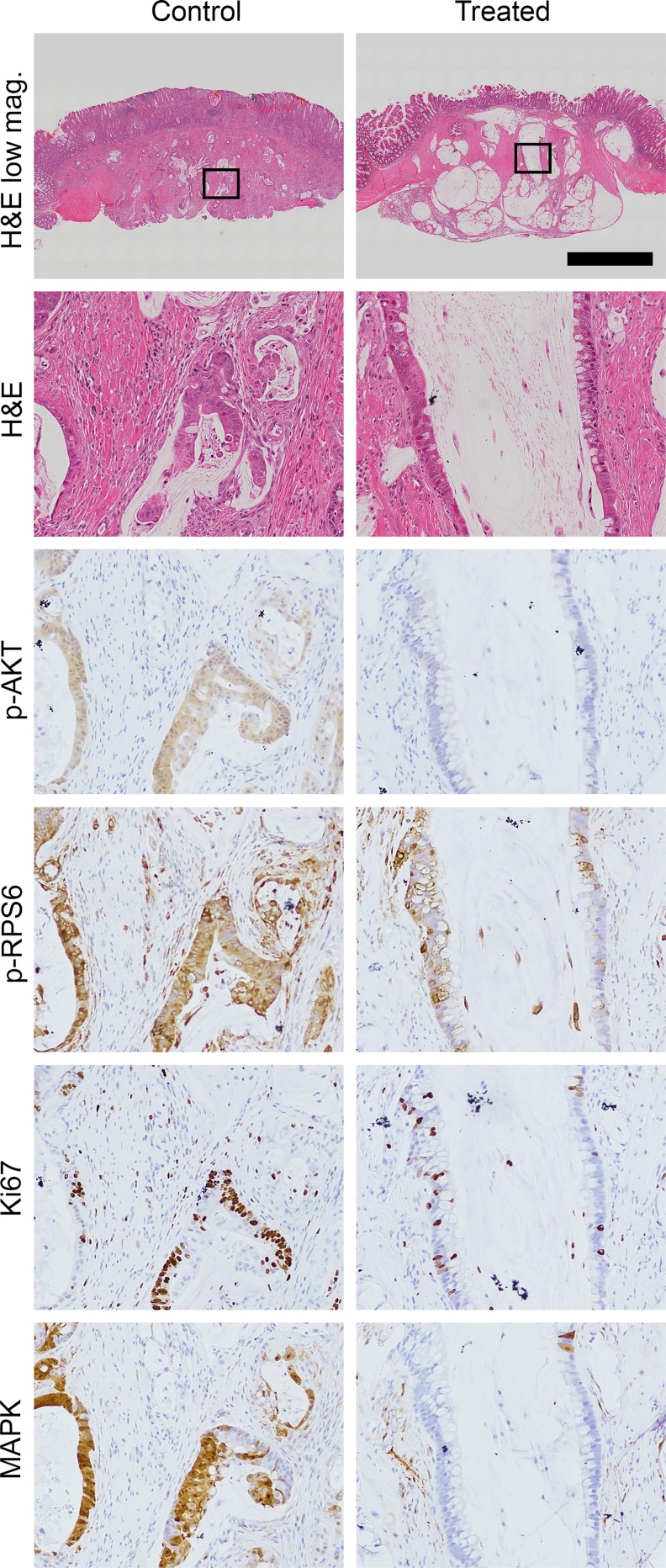
Histological examination demonstrates a significant treatment effect following dual PI3K/mTOR inhibition of *Fc*^*+*^ *Pik3ca*^*H1047R*^ cancers. In comparison to controls, a dramatic treatment effect was identified on H&E staining: loss of epithelial cells surrounding the areas of cystic change/degeneration and areas of increased fibrosis. A decrease in Ki67 staining and phosphorylation of AKT, RPS6 and ERK1/2 are also seen. Scale bar for low magnification images = 1mm. High magnification panels are 10x magnifications of the areas indicated by the rectangles.

Following imaging, the mice were treated again with NVP-BEZ235 or control and necropsy performed 1 hour later. At necropsy, 18 tumors were identified in control mice compared to 12 in *Fc*^*+*^ *Pik3ca*^*H1047R*^ mice treated with NVP-BEZ235. No significant difference in median cancer size was observed (3.6 vs 3.2 mm, p = 0.59). Tumors were fixed in formalin and embedded in paraffin. H&E staining demonstrated a significant treatment response in those tumors treated with NVP-BEZ235, including a loss of epithelial cells surrounding the mucin lakes areas of cystic change/degeneration and areas of increased fibrosis, consistent with treatment effect (Fig 6). Dual PI3K/mTOR inhibition resulted in decreased phosphorylation of AKT and RPS6 (Fig 6). Decreased proliferation as measured by Ki67 staining was observed (Fig 6). Additionally, the phosphorylated ERK1/2 is also diminished with NVP-BEZ235 treatment (Fig 6).

## Discussion

The PI3K pathway remains a target of interest for the development of novel therapeutic strategies for many cancers given the importance of this pathway for multiple vital cellular functions [18]. It is one of the most commonly aberrant pathways in cancer [7]. PI3K delta inhibitors, such as idelalisib, have been successfully applied to the treatment of hematologic malignancies [19]. In solid tumors, the alpha and beta isoforms are the predominant mediators of PI3K signaling, with minimal contributions from the gamma or delta isoforms. Response to PI3K alpha selective and pan inhibitors has been observed in certain settings [20]. Multiple early phase clinical studies are investigating the use of these agents in the setting of *PIK3CA* mutations [21–23]. Recently, in a phase I clinical trial a partial response was seen in a colorectal cancer patient treated with LY3023414, a dual PI3K/mTOR inhibitor [24]. However, the contribution of the *PIK3CA* mutation to the biology of these cancers has been under-investigated and the population of patients most likely to benefit from these agents has yet to be determined. This is especially true for colorectal cancer where *PIK3CA* mutations are most commonly thought to be a late event in tumorigenesis and the potential benefit from PI3K pathway inhibition is uncertain.

Only recently have the effects of a dominant active PI3K pathway been explored in the mammalian intestine. Our group was the first to describe the development of hyperplasia and invasive mucinous adenocarcinomas developing in the proximal colon as a result of expression of a dominant active PI3K (*Pik3ca*^p110*^) [10]. The cancers in this model developed through a non-canonical pathway without the identification of a polypoid intermediary and without activation of WNT signaling. To determine if similar tumors could be initiated in the setting of a hotspot mutation in *PIK3CA* commonly encountered in human cancers, we developed the *Fc*^*+*^ *Pik3ca*^*H1047R*^ murine model. Here we demonstrate that the *Pik3ca*^*H1047R*^ mutation results in hyperplasia and the development of mucinous adenocarcinomas within the colon indistinguishable from the *Fc*^*+*^ *Pik3ca*^p110*^ mice. The predominant difference between these two models is the latency in which these tumors develop. In the *Fc*^*+*^ *Pik3ca*^p110*^ model, ~75% of mice had invasive cancers by 40 days of age [10], while in the *Fc*^*+*^ *Pik3ca*^*H1047R*^ mice no tumors were identified at 50 days of age and only 65% of mice had tumors at necropsy (age 97–310 days). These cancers appear to be developing through a similar mechanism with activation of the PI3K pathway and without activation of WNT signaling. The increased latency in cancer development in the *Fc*^*+*^ *Pik3ca*^*H1047R*^ mice appears related to a decreased level of activation of the PI3K pathway compared to the *Fc*^*+*^ *Pik3ca*^p110*^ mice.

Interestingly, increased ERK1/2 phosphorylation was observed in both the *Fc*^*+*^ *Pik3ca*^p110*^ and *Fc*^*+*^ *Pik3ca*^*H1047R*^ tumors compared to the *Apc*^*Min*^ colon tumors. In these cancers it does not appear to be a mechanism of resistance to PI3K pathway inhibition, though this has been demonstrated in some settings [25, 26]. Activation of ERK1/2 signaling has been observed in other models with *PIK3CA* mutations including *PIK3CA* mutant breast and pancreatic cancers [27, 28]. Activation of ERK1/2 seems to be largely independent of RAS in these *Pik3ca* mutant tumors [27]. It is instead potentially mediated by the RAC1/CRAF/MEK/ERK pathway. The phosphorylation of ERK1/2, in the models presented here, is likely downstream of PI3K signaling, as it is diminished with PI3K/mTOR inhibition.

We hypothesize that there is a subpopulation of patients that have cancers initiated by or dependent upon the PI3K signaling cascade. The cancers in this subpopulation may be extremely susceptible to many of the novel PI3K inhibitors similar to NVP-BEZ235. In a recent pathological series, activating mutations in *PIK3CA* were observed more commonly in mucinous colon cancers in humans, similar to our model, and were associated with worsened prognosis [29]. Interestingly, in this same investigation, activating mutations in *PIK3CA* were inversely associated with the nuclear translocation of β-catenin [29]. Translocation of β-catenin would be expected if these tumors were initiated by aberrant WNT signaling as part of the previously described canonical mechanisms of tumorigenesis in colon cancers [30]. Together, these observations indicate that a subgroup of human colon cancers might arise in the setting of activated PI3K, as seen in our model.

## Supporting Information

S1 Fig Additional examples of baseline and post-treatment 18F-FDG PET/CT scans of *Fc*^*+*^ *Pik3ca*^*H1047R*^ mice treated with NVP-BEZ235 (35mg/kg/day) or control once daily by oral gavage for 14 days. Arrows denote tumors pre- and post-treatment.
(PDF)

S1 Table Characteristics of *Apc*^*Min/+*^, *Fc*^*+*^ *Pik3c*a^p110*^, and *Fc*^*+*^ *Pik3ca*^*H1047R*^ mice. (PDF)

S2 Table Pretreatment volume of tumors imaged with PET/CT. (PDF)
