# Supplementary figures and images for: Colon Cancer Tumorigenesis Initiated by the H1047R Mutant PI3K
Source: PLoS One. 2016 Feb 10;11(2):e0148730. doi: 10.1371/journal.pone.0148730 (PMC4749659; doi:10.1371/journal.pone.0148730)

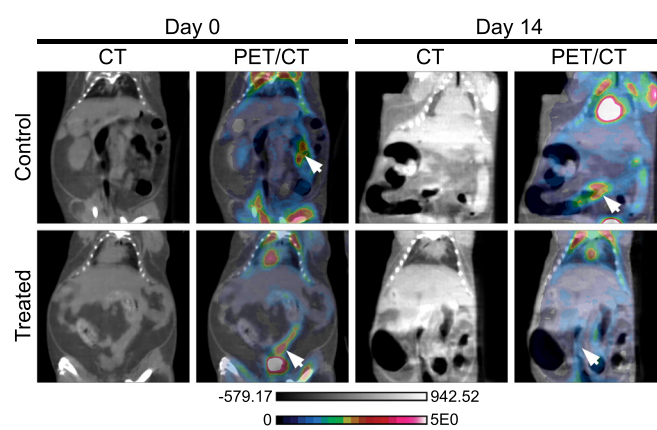

Supplement: S1 Fig — Arrows denote tumors pre- and post-treatment. (PDF) [file pone.0148730.s001.pdf]
