# Supplementary material for: Colon Cancer Tumorigenesis Initiated by the H1047R Mutant PI3K
Source: PLoS One. 2016 Feb 10;11(2):e0148730. doi: 10.1371/journal.pone.0148730 (PMC4749659; doi:10.1371/journal.pone.0148730)
Supplement: S1 Table — (PDF) [file pone.0148730.s002.pdf]

S1 Table. Comparison of mouse models.

Mice	Small intestinal tumor count	Colon tumor count	Tumors progressing to cancer	Lifespan
C57BL/6J *Apc*$^{Min/+}$	92 ± 4 (males)<br>103 ± 4 (females)[a]	2.9 ± 0.2 (males)<br>1.6 ± 0.1 (females)[a]	0%[b]	Rarely survive beyond 120d[c]
(FVB x B6)F1 *Fc*$^{+}$ *Pik3ca*^{p110*}	None[d]	1.4[d]	100%[e]	Majority moribund at 40-60d[e]
FVB *Fc*$^{+}$ *Pik3ca*^{H1047R}	None[f]	1.2 ± 1.4[f]	100%[f]	>150d[f]

[a]Amos-Landgraf JM et al., Proc Natl Acac Sci USA 2014;111:16514-9. Data shown as mean ± SEM.

[b]Halberg RB et al., Cancer Res 2009;69:5768-75.

[c]Moser AR et al., Science 1990;247:322-4.

[d]Deming DA et al., Oncogene 2014;33:2245-54. Colon tumor count presented is the mean.

[e]Leystra AA et al., Cancer Res 2012;72:293106.

[f]This study. Colon tumor count shown as mean ± SD.
