# Supplementary material for: Colon Cancer Tumorigenesis Initiated by the H1047R Mutant PI3K
Source: PLoS One. 2016 Feb 10;11(2):e0148730. doi: 10.1371/journal.pone.0148730 (PMC4749659; doi:10.1371/journal.pone.0148730)
Supplement: S2 Table — (PDF) [file pone.0148730.s003.pdf]

S2 Table. Pretreatment volume of tumors imaged with PET/CT.

Treatment group	N of tumors	Pretreatment tumor volume		
		Mean	Median	Range
Control	13	30.6	21.6	3.2-106.9
Treated with NVP-BEZ235	11	42.3	30.4	3.9-88.0
